# Implementation Science for Managers and Healthcare Organizations Responding to Emergencies

**DOI:** 10.1007/s43477-021-00025-0

**Published:** 2021-10-21

**Authors:** John Øvretveit, Mikael Ohrling

**Affiliations:** 1 Medical Management Centre, Department of Learning Management Informatics and Ethics, Karolinska Institutet, and Research and Development Officer, Stockholm Health Care Services, Stockholm, Sweden; 2 Stockholm Health Care Services, Stockholm, Sweden

**Keywords:** Implementation science, Disaster management, COVID-19, Primary and community healthcare, Action research, Partnership research

## Abstract

The purpose of the article is to illustrate how implementation science concepts and methods can be applied by researchers and implementers to understand and assist emergency management in a large primary and community healthcare organization. The article refers to a single-case implementation action evaluation of an emergency management system in a healthcare organization. It describes the methods used in this study and findings to explain how a joint healthcare and university research team were able to use the science and methods both to help implementation and contribute to science. We report two sets of findings. First, findings about implementation of emergency management to illustrate how the investigation adapted implementation science and concepts to achieve the objectives evaluation. We discovered that implementation science provides useful concepts to understand contextual factors and adds to knowledge about organizational change and emergency management in the uncertain and evolving situation we encountered. The second set of findings are the strengths and limitations of both implementation science and the action evaluation methods we used to achieve the dual objectives of practical help with implementation and to contribute to science. The article uses the first implementation action evaluation study of the response of large public primary and community healthcare organization to a pandemic to illustrate how implementation science can be applied. This type of study was able to improve implementation of the response as well as contribute to scientific knowledge about emergency healthcare management and organization.

In 2020, many healthcare managers made more changes to their services than they had made in the last 5 years. They made these changes quickly to respond to the COVID-19 pandemic, and changes often had to be revised. Their skills and stamina, and those of clinical staff were tested to the limit, and are still being tested. Far from their minds was the relatively new science of implementation. Implementation researchers considered what they could do to contribute. Our understanding is that many found their science and research methods to have limitations for enabling healthcare managers and their organizations rapidly to respond to a pandemic.

Much is being learned by researchers retrospectively about the implementation of evidence-based practices and service delivery models during the pandemic that will be useful in the future. However, the focus of this article is on how implementation science can be used to enable more effective and faster change to respond to evolving crises, where evidence may be limited or changing, and adaptation and fast adjustment are needed. For some of the global readership of this journal, this may be the situation now, and with more variants and infectious diseases, this may be the situation for all of us in the future.

This article first notes relevant literature about change management and emergency planning and disaster management. Then it describes the methods we used in our investigation. We do not describe all the details of the methods and findings because the purpose of the article is to show, through this example and our experience, how implementation science (IS) can be used to help implementation and carry out rapid research during a pandemic or evolving emergency. We describe details of the method and findings elsewhere (Ohrling et al., 2020; Øvretveit, 2020a). We then discuss what we learned about applying IS for emergency management implementation and research that others could use, and value of implementation action evaluation to the field of emergency and disaster management.

## Knowledge Potentially Relevant to Implementing an Emergency Response in Healthcare

There is a large literature about both managing change and emergency management and organization, and some is based on research (CDC, 2018; FEMA, 2011; Iles & Sutherland, 2001; Koenig & Schultz, 2009; Rose et al., 2017). Most research is about single-event emergencies, rather than evolving crises with many different dimensions like the SARS COV-2 pandemic. Research into the developing response to HIV AIDS is perhaps the exception (Shilts, 2007). UK studies of emergency planning and management in health care did not consider implementation science or implementation to be a priority research topic (Boyd et al., 2014; Lee et al., 2012). There is little empirical research into changes in the operations of service delivery organizations in emergencies, especially in primary and community services. This article describes the methods and findings we used in our study to give illustrations of implementation science concepts that we found useful for evaluating an organization and management response to an evolving crisis.

## Background to Applying Implementation Science in Stockholm Healthcare

During 2020 and 2021, Stockholm region healthcare rapidly implemented many changes to healthcare service delivery to respond to the SARS COV-2 pandemic. In early March 2020, we formed a research team to document and evaluate the management and organizational changes made by one organization within the region to respond to the pandemic. This was the single organization that managed all the public primary and community healthcare (P&CHc) services in Stockholm, called the Stockholm County Healthcare Area, or SLSO in Swedish. These include a diverse range of 120 service delivery units (SDUs) including, 70 primary care centers; specialist “hospital at home” and palliative care; services for psychiatry, addiction, rehabilitation; and 2 large community-based geriatric hospitals (SLSO, 2020). We refer to the senior management of this organization as the P&CHc SLSO management.

The health and social care services in Stockholm region are tax-funded and provided by a mix of public and private health and social care services for the 2.3 million population. Sixty percent of primary care facilities are private, as well as one acute hospital and 12 geriatric hospitals, but these private services are publicly-financed and regulated. The 26 municipalities in the region provide health-related services or purchased these, including non-medical services for older people, such as private home visiting services and care homes for 15,000 people. The municipalities are small independent local authorities and separate from the Region Stockholm county government.

### The Implementation Evaluation of the Response

The objectives as stated in the research protocol are,

To describe and evaluate the implementation of changes made to the organization and service operations of the Stockholm primary and community healthcare service to respond to the Covid-19 virus. The purpose of doing this is to improve the emergency response of this service during the outbreak, and then to help plan a better response for future contagious diseases of this type, as well as to provide scientific knowledge for the emerging discipline of emergency management and implementation science for emergency response (Øvretveit, 2020a, p. 2).

We started the research in mid-March 2020, continuing into 2021, by a combined team of six researchers from the Karolinska Institutet medical university and researchers and administrators employed by Region Stockholm and working in the P&CHc SLSO organization. The authors of this article made a joint ethics application that was approved in early April 2020 (Region Stockholm Dnr 2020-01,521, 2020).

To achieve the purposes of this article, we reference research methods and data that were collected in the first part of 2020, and published in Ohrling et al., (2020), to illustrate how we used IS. We refer to other data, not yet fully analyzed, to illustrate, for example, the continuous adaptation of the innovation we are evaluating. We use these illustrations later to discuss the benefits and limitations of IS and of our investigation approach for the two objectives of the investigation: practical help and scientific contribution.

## Methods

### Research Design: Choice and Challenge

**Fig. 1:**
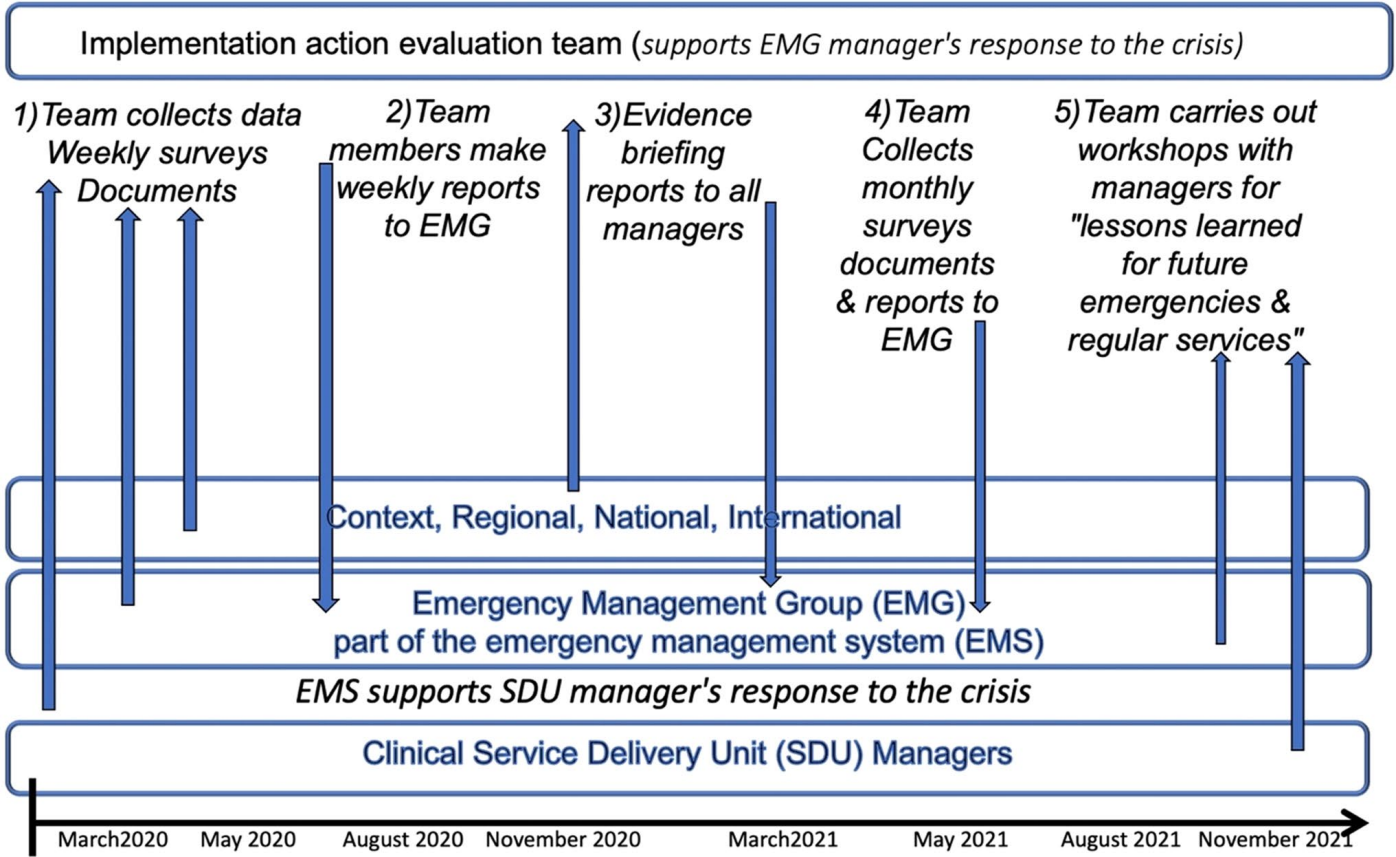
Design of the implementation action evaluation of emergency management in a primary and community healthcare service delivery organization

We chose a single-case implementation action evaluation design using mixed-methods and a participatory research approach. Our challenge was to find or create a design, data collection and analysis methods that provide the most valid data and conclusions for the dual practical and scientific aims, but were feasible and timely, given the limited resources available for the investigation (Fig. 1).

We consider part of the design to be the concepts that we used to decide which data to gather and to analyze the data. The methods section sub-headings correspond to the sub-headings in the results and discussion sections. We give more details about in other publications and in the research protocol for the investigation (Ohrling et al., 2020; Øvretveit, 2020a).

### Methods for Documenting Context and Understanding Influence

IS research shows that the implementation, outcome and nature of innovations are influenced by certain features of the context (Nilsen & Bernhardsson, 2019). To decide which data to collect, we used operational specifications of inner and outer context (CFIR, 2021). We collected data about context, and about the other subjects described later, from four types of sources. First, from documents that included minutes of each emergency management group meeting (*n* = 84); a diary made by the principle investigator and first author, that summarizes national and regional directives, guidance and changes made from public agencies and from regular regional guidance issued to healthcare providers; and plans for an emergency response (Engström & Ohrling, 2019).

Another source was interviews with all 15 members of the SLSO emergency management group (EMG), performed by four of the implementation action evaluation investigation team during May and June 2020, and monthly interviews with the CEO of SLSO throughout 2020 and 2021. We used a semi-structured interview guide, and recorded and transcribed the interviews. The third source was a nine-question weekly survey of the 120 managers of the SDUs in the SLSO P&CHc organization, with free text sections for additional comments. We using the internal SLSO secure internet with the SLSO CEO’s invite to complete the survey to administer the survey. SDU managers completed it on-line and anonymously. The responses were quickly analyzed and reported to the SLSO EMG to give them an overall view of the managers’ situations and provided data for the evaluation team to track one outcome of the implementation of the EMS. In 2021, we issued the survey every month. The fourth source was the Region’s data bases which provided data about the number of people testing positive for SARS COV-2 infection, and admitted to hospital or intensive care unit for COVID-19 illness, as well as data from a regular simulation modeling that make predictions about future infections and admissions.

We analyzed data about context and the other subjects using two main approaches. We analyzed qualitative data inductively, by categorizing themes for each interview and then reconciling different interpretations among the team (Miles & Huberman, 1984). We used a deductive approach applying definitions of context in the CFIR 2021 guide to examine whether these were mentioned as influential in the interviews or documents. We used the survey software provided for the internal surveys to analyze the quantitative data from the weekly surveys.

### Methods for Documenting the Innovation and Its Implementation

There are no standardized concepts or operational specifications for collecting data about an emergency management innovation and its implementation. Thus, we formulated the following definitions to guide our data collection: we defined emergency management in healthcare service delivery organizations as the organization and management of the resources and responsibilities and the implementation of changes for providing healthcare in response to an emergency event or episode. It includes responses to the specific emergency event or episode, as well as prioritizing and providing routine healthcare that was provided before the emergency. It includes managing the balance of resources and responses between the emergency and routine care at different phases of the emergency episode.

We defined the innovation made to the P&CHc SLSO organzation as the emergency management system (EMS), with the SLSO senior emergency management group (EMG) as a key part of the structure of the EMS. We defined implementation as the actions and strategies used by the EMG to enable their and others’ uptake of the EMS innovation in the SLSO daily activities (e.g., new roles they performed in the EMG), in support systems (e.g., new data systems and human resources practices) and in organization (e.g., new work practices and relationships in the SLSO central management unit).

The data collection sources to document the EMS innovation and implementation actions included the emergency management plan (was this followed?), and the other sources described above, apart from the fourth sources of statistical data. The analysis methods were the same as those used to analyze data about context.

### Methods for Collecting and Analyzing Data About Outcomes

The management level below the senior managers in the EMG is the 120 managers of the primary and community healthcare SDUs. We used IS concepts to define the outcomes of the implementation of the EMS innovation to be the difference this made for the ability of the SDU managers to respond to the pandemic, compared to what would have happened if there had been no EMS innovation implemented within the changing context.

We used data from responses to some questions in the survey to SDU managers to make an estimation of this outcome of the EMS innovation. Are there any new rules or instructions that you must follow that made it easier or more difficult to respond to the Covid-19 outbreak? What help do you most need urgently from SLSO management to minimize the spread of infections or morbidity/mortality? Examples of questions are, do you need help from the SLSO leadership to maintain the health of your personnel? Compared to your normal staffing (100%) this week, did you have on average more, or less staff than normal as a percentage out of 100% (Øvretveit, 2020a)? We also used data from the interviews with the 15 SLSO EMG members and the monthly CEO interviews to gather subjective impressions of the difference the EMS may have made, compared to keeping the way of working in 2019. Both these types of data and the single-case research design with no comparison case gave limited and uncertain data about implementation outcomes: we consider this issue in the discussion section.

### Methods for Implementation Action Evaluation

The implementation independent variable is the reporting carried out by the researchers in the action evaluation. The dependent variables are the actions of the EMG to create change in the health system. Possible changes are to enable implementation of the EMS and modifications to the EMS innovation to be more effective, as well as to provide information about context factors that could inform EMG decisions, such as about possible future outbreaks. The results section describes findings reported to the EMG about context (e.g., infection simulation prediction, as well as evidence updates from international experience), and about support the SDU managers reported they needed from the EMG in the weekly surveys. As regards the methods for feedback, this was presentations to the EMG weekly or as invited, by two members of the investigation team about the survey and timely infection feedback. Also in 2021, briefing summaries were provided giving relevant evidence and overseas experience (Øvretveit, 2021).

## Results

### Findings About the Context for the SARS COV-2 Outbreak in Stockholm

Findings regarding the international context, shown in the published literature are: the evidence of rapid spread of the virus and its variants (Riggioni et al., 2020), the rapid availability of changing and sometimes confusing evidence from research and statistical sources (CDC, 2021) and the large number of items published on the internet used by patients and citizens, much of which is unreliable or misleading (Cuan-Baltazar et al., 2020).

One finding from the documentation of the context for the implementation of the EMS is that Sweden was unusual in not instituting a “lockdown” or restricting freedom of movement in the first 8 months of the pandemic. The advice of the national public health agency was to wear a mask, to observe 2 m physical distance and hand hygiene. There was no significant contact tracing program. In November 2020, the national and regional government introduced some laws against large gatherings for time-limited periods, due to a rapid rise of winter infections. Overall, the infection and death rates were slightly less per capita than the UK, with its three major “lockdowns,” and followed the same trends as the UK over the entire period, but were much greater than the other four Nordic countries. The causes of these similarities and differences are, and will be, much debated (Klein et al., 2020).

Documents and interviews show that the national context of laws and regulations, as well as guidance by the Sweden public health agency, changed little during 2020. However, during 2021, with the increased spread resulting from the more infectious delta variant of the virus, the guidance changed to align more closely with other European countries. The Government emphasized its advice to work at home where possible, and about testing, and introduced new laws about the maximum number of people allowed in shops and sports facilities, and a maximum limit for all events.

Regarding the regional context, statistics show that virus spread rapidly in Stockholm in March 2020 and the hospitals stopped elective- and most non-covid services. Many SLSO managers reported factors related to delays or inaction by the regional organization in March and April 2020 that hindered the EMG’s ability to operate the EMS and to implement the emergency plan. The NATO emergency management approach was to centralize all decision-making to the regional emergency management group (REMG) to ensure coordination, and to require the lower level EMGs in the hospitals and SLSO to refer key decisions to the REMG for authorization. The SLSO interviewees described two problematic contextual factors: limited or no protective personal equipment and lack of timely information. Our findings about the innovation and its implementation follow and detail some of the actions taken by the EMG to respond to these challenges."

Some EMG interviewees also reported that an enabling context internal to SLSO was the previous 15-year management decentralization program. Two interviewees reported this decentralization developed the capacity of the clinical level SDU managers to make and be held accountable for decisions, such as staff mix and expenditures up to 5000$. In addition, that it developed a culture and systems for maximizing decentralization but allowing coordination to avoid each unit optimizing their performance at the expense of others and of overall performance of the health system (Ohrling et al., 2021). Our findings thus may be more likely to generalize to health systems that have a positive history of decentralized decision-making and accountability.

### Findings About the Innovation and Its Implementation

Minutes of the Region Stockholm government and data from interviews with SLSO EMG members showed that, on February 29th 2020, the Region instructed the CEO of the P&CHc SLSO division and the other regional public organizations to activate the service’s crisis plan. This required forming of a senior level emergency management group in the SLSO division, and an EMG in each hospital, all reporting to the regional level EMG, as well as an emergency management system (EMS) to carry out the 10 functions of the NATO emergency management model (Engström & Ohrling, 2019; Granåsen & Olsén, 2019).

The SLSO EMG members interviewed reported that this was the first time the region and this service division had implemented the emergency plan or had ever faced a fast spreading and widespread novel infectious virus, about which little was known. As regards implementing the NATO management system, the first item reported by interviewees was challenges experienced by senior SLSO managers and others performing the new functional role that was required of those appointed to the SLSO EMG of the NATO management system. Many reported that it took time to learn the role, and that they needed time to form an effective way of working as a group. This involved trying different procedures for what members described as overly long meetings, and learning how all the functions and roles were needed and learning to work effectively as a group. Interviewees reported that modifications were made continually over 18 months, including problem solving sub-groups delegated by the EMG to work on an issue that needed more time, and then to report back to the EMG main meetings.

Interviewees reported that the operation of the planned NATO management system by the region in the first weeks resulted in delays in decision-making, with the EMS requiring most decisions to be referred to the regional emergency management group. Actions taken by the SLSO CEO and the heads of hospitals were to “exert pressure upward” by making frequent requests to the REMG to make decisions, especially about under-supply of protective personal equipment (PPE) by the regional supply unit and a lack of timely information about infections, admissions and intensive care unit (ICU) capacity.

As regards other findings about PPE early in the outbreak, the weekly 9-question survey to all 120 SLSO first-level managers of the SDUs gave additional evidence of the PPE shortages as the primary and most urgent problem that these managers faced, and of how widespread this was across the region. Findings from the survey were reported to the SLSO EMG 4 days after the managers' responses. These data, and what some SLSO EMG members had heard from SDU managers, revealed that the regional-managed purchasing and supply unit was not able adequately to purchase and deliver PPE. Interviewees reported that, early in April 2020, the combined CEOs of the SLSO and hospitals group decided to “take the matter into their own hands” and used an opportunity and personal contacts quickly to set up a special PPE unit. This was jointly staffed by their employees and a construction company purchasing and logistics staff, and housed in the hospital next to the SLSO head office. The staff purchased and flew in PPE quickly because of their trusted and global reputation. The SLSO head offices and other facilities stored and distributed PPE to their and other SDUs using a variety of voluntary and formal delivery arrangements. They also set up a computer system for SDUs to order and track deliveries, all separate from the regional unit. The research team defined this as both an adaptation to the structure of the EMS innovation, and an adaptation to the implementation methods. The success of this adaptation led to the Region formalizing this arrangement and giving SLSO senior management oversight of the system. We recognize that this is not easily generalizable, but is an example of resourcefulness, taking opportunities, the collective action that is easier in a crisis, and the attitude of asking for forgiveness later if it saves lives and does no harm.

The second problem early in the outbreak reported by interviewees was inadequate information about infections, hospital admissions, and ICU admissions in relation to capacity. Normally a regional office provided this, but the group of CEOs from SLSO and hospitals needed more timely information, as well as predictive modeling for decisions and planning. Interviewees noted that cooperative planning between the SLSO EMG and hospitals became very important to support people at home to reduce unnecessary admissions and maintain capacity in hospitals. This led to two members of the implementation action evaluation team, who were also members of the SLSO EMG, collecting local data and providing data analysis reports every week to the SLSO EMG and hospitals about infections, admissions and ICU occupancy and also working with a hospital analytics unit using an open source predictive modeling tool. This data analysis could be considered part of the implementation action evaluation team feedback, or it could be viewed as an adaptation to the EMS and its implementation, or both.

The above results refer to the implementation action evaluation findings about details of the context, the innovation (the SLSO EMS) and its implementation as conceptualized using IS concepts. The evaluation findings contributed to two key adaptations related to improved PPE access and distribution, and to timely local data related to infections, admissions, and ICU occupancy. The findings about these subjects after April 2020 from interviews, documents, statistical data and surveys show continuing changes to context as well as adaptations to the EMS innovation and its implementation, but none as significant as these early changes. Perhaps the most important findings for later in 2020, when infections declined, concerned how the SLSO EMS then attempted to support the SDUs in restarting non-COVID-19 services and manage these parallel to the COVID-19 services. We do not report these findings here because we are still analyzing them, and because the aim of the article is to show how IS can be used in a rapid implementation action evaluations rather than provide a fully comprehensive research report.

### Findings About Implementation Outcomes

The proximal outcomes from implementing the SLSO EMS innovation would be the changes that SDU managers and staff were able to make to the service, that they would not have been able to make without the EMS implemented. Data from the weekly surveys in May 2020 show one outcome was a fast reduction of PPE supply problems, which SLSO EMG interviewees attributed to the new purchasing and logistic service overseen by the SLSO EMG. Other survey data show that most SDU managers agreed that the SLSO EMG had provided strong support. Another outcome described in the interviews and minutes of meetings of the SLSO EMG is the formation by the EMG of three coordination groups in different areas of the region. The SLSO EMG invited heads of healthcare services from SLSO, private services and heads of social services run by smaller local authority municipalities and delegated local coordination of each to a SLSO manager. Interviewees reported that these voluntary, active and effective groups would not have been formed without the EMG initiation and that the shared context of together facing the COVID-19 emergency gave a motivation to work together in the crisis.

### Findings About Later Implementation of the Innovation

The interviews and SLSO EMG documentation show that in May and in June 2020 the twice-daily meetings of the emergency group were reduced to once a day and then three a week. After 10 July, the SLSO emergency management system was dis-established, and ‘normal operations’ declared on 10 July 2020. These data showed that this de-implementation was in response to two context changes: a dramatic reduction in infections and disease activity and regional government guidance. However, interviews revealed that a modified version of the EMG with a smaller group and weekly meetings continued, and was ready to meet more frequently later in the winter.

Later findings in November 2020 from the surveys and interviews with the SLSO CEO showed that a second outbreak was beginning and the modified EMG increasing its frequency of meeting, before the region directed a higher level of alert. Interviews with the SLSO CEO suggest that the implementation action evaluation team reporting evidence from the UK about the impact of the more infectious alpha SARS COV-2 variant, may have influenced this, as well as the reporting about local infection increase that showed the second major outbreak during January and February 2021.

In summary, the findings about implementation of the innovation over an extended 18-month period from March 2020 to August 2021 showed not so much a linear set of stages of implementation but a continual adaptation of both the EMS and its implementation, in response to aspects of the context such as the rise and fall of the two major outbreaks in March/April 2020 and January/February 2021. Some interviewees of the EMG reported that adaptations were assisted in a small part by feedback from the implementation action evaluation team, but we could not precisely assess the effect of this feedback.

### Findings About Implementing Innovations with No- or Uncertain- Evidence

The third main theme of the SDU managers’ survey responses, especially early in the outbreak, was not having sufficient information. The fourth was having too much information from emails and other sources. In the free text replies, some managers wanted more evidence about protection of infection and the current risks for staff and for patients.

Some of the SLSO EMG members interviewed reported that the EMS had never been implemented in SLSO, and that there was no evidence about the effectiveness of an EMS modeled on the NATO emergency system in healthcare service delivery organizations. Two commented that perhaps the implementation action evaluation could provide some evidence.

Four other related themes emerged from the analysis of these interviews, and from the surveys and documents. The first was that all managers appeared to pay more attention to local data and research evidence and to questioning whether the evidence about a possible change that was suggested was strong enough to justify implementation. For example, early in the outbreak, few questioned the evidence about wearing masks and other PPE, but later as supplies became available and staff found the time and inconvenience of using PPE burdensome, more asked for specific evidence about the protection of different masks in different situations and periods. The research team later was able to provide more of this evidence, but it is unclear how much this affected practice or the directives of the EMG to SDU managers.

The second theme, perhaps conflicting with the first, was that knowledge that “makes sense” to managers, especially if related to their experience, appeared to be stronger to their motivation to implementing changes than research evidence about the change, even when there was evidence. For example, one EMG manager interviewed commented that “if it is a respiratory virus like influenza, then keeping out of the way of people sneezing and 2-m distance makes sense to me and my staff. I do not need research evidence to implement this in my clinic”.

The third theme concerned the changing amount and certainty of evidence. Some built on or corrected earlier evidence, such as new evidence that aerosols as well as larger droplets also carried the virus, or about asymptomatic people spreading the virus, or about different transmission of virus variants. Many EMG managers reported how confusing this was, and not knowing when new evidence should cause them to change their directives to staff.

The fourth theme was of continued uncertainty about identified issues, and discovering issues that raised more uncertainties. This theme was exemplified by one manager's comment stating the challenge of "not knowing what we want to know and not knowing what we don't know we don't know". Examples were, in March 2020, of not knowing how long the emergency would last, how many staff would be off sick, and if a vaccine could be developed. No one imagined Long Covid or its impact on primary care, and, in 2021 effective curative treatments are still unknown. When early trials showed vaccines may be effective, we did not know for some time when they would be available and or about evidence to decide who should be vaccinated first. These themes show that the EMG and SDU managers had never faced so much continued uncertainty about so many high-consequence matters that they had to make decisions about, and with so little or changing evidence.

## Discussion

### Research Challenges

One research challenge we faced at the start in March 2020 was how best to use implementation concepts to guide our data collection to evaluate the emergency response to the pandemic. This relates to the question of which research design to use so that we could attribute outcomes to the innovation and implementation, rather than to some other influence.

Another challenge was that we had limited resources and could not evaluate all the changes and their outcomes in the many service delivery units. We could not consider each change that both the senior and the 120 SDU clinic managers needed to make at different times as separate innovations (for example, to change the physical layout and people flow in clinics to enable distancing, as well as other changes at later times).

Our research aim was to provide timely feedback to management to enable them to adjust their support to clinic level providers, as well as to contribute to science. Our initial search could not find any relevant research to build on about emergency management implementation in service delivery organizations, let alone in a large public primary and community healthcare organization. Would more time searching provide us with a suitable design and guidance for data collection from previous research? Were there suitable data-banks that could provide us with timely and accessible data about different subjects relevant to our study, so that we would not need to create our own data collection systems? It was a challenge to balance competing needs related to data selection and collection. There was an urgent need to begin data collection about implementation. But we also needed time to plan so that data would be useful for managers, would advance the science of implementation, and would avoid wasting time collecting data that would not be used. This discussion considers some of the ways we addressed these challenges, the limitations of our resolutions and the possible contributions we made to methods for implementation action evaluation and to the developing science of emergency management and organization.

### Context for the SARS COV-2 Outbreak in Stockholm

We found a research based IS study that gave clear operational definitions for the concept of context useful for our study (Damschroder et al., 2009; CFIR, 2021). This specifies elements of inner and outer contexts and this allows more valid comparisons with other studies to see if they found these features important when studying the innovation and implementation that they considered. For example, SLSO management reported outer context factors of regulations and finance to be a key driver for their implementation of the EMS innovation. Greenhalgh et al. (2004) also report these specific elements to be important influences of diffusion of innovations in service organizations, and this study is also referenced as evidence in the CFIR 2021 guidance for this element of context.

### Innovation and Implementation

Notwithstanding the above useful IS study, we did find a limitation of IS for our study to be the different definitions of implementation science concepts, and a lack of operational definitions or measures to guide data collection (Brownson et al., 2012). An example is the definition for the new better way of working or organizing that is to be implemented in daily practice (Øvretveit, 2014). The convention in healthcare is to call this an intervention, but there are different definitions of this term and, for some, it may imply a discrete time-limited action, such as a surgical procedure. In many sectors outside of healthcare the convention is to call this an innovation, but also with different definitions (Fixsen & Fixsen, 2016). For the wide readership of this journal, we chose the term, innovation, and made the definition we gave earlier in the methods section. Because we were evaluating the EMS, we did not consider specific innovations to enable individual behavior change. However, we found useful, when advising the management team about alternative evidence-based behavior change strategies, the IS taxonomy of 93 behavior changes (Michie et al., 2015). This illustrated to others the different ways, apart from training, that could be used to enable clinic staff and others to uptake new ways of working, and sometimes helped us to communicate the changes reported in the surveys by clinic mangers of SDUs.

As regards implementation, the results documented issues and approaches in implementing the EMS innovation, including staff in the EMG learning new roles, changing procedures and forming sub-groups. We found it useful to apply deductive analysis using a taxonomy of 73 implementation strategies to clarify, classify and communicate different implementation strategies (Powell et al., 2015). For example, strategy 1 “Access new funding” was used early to fund the changes to implement the SLSO EMS, as was strategy 11 “Change physical structure and equipment” at the SLSO central office, including PPE storage for distribution, and strategy 15 “Conduct educational meetings”. As regards later forming the three area multi-sector coordination groups as part of the EMS, this involved both strategies 6 and 7: “Build a coalition”, and “Capture and share local knowledge “. The EMS implementation also involved strategy 67 “Use data experts” including using SLSO staff as well as the research evaluation team.

### Outcomes

We found useful the IS concepts distinguishing the proximal outcomes of implementation from the more distant service changes, and then these from possible later changes in patient health outcomes and experience (Proctor et al., 2011). Applying this in our study, we considered what “comes out” of the implementation actions that applied the innovation change (the EMS). We defined the immediate outcome of implementing the emergency management system as the impact the EMS had on the work that clinic level service delivery managers needed to make to change work practices and organization of their units. The results section illustrated this with findings about the initial outcomes of the EMS. More challenging was conceptualizing outcomes of the modified and continually changing EMS that was shown in the data in the results section, and how to document the changes and report these over time. Another challenge was to assess how much, if at all, the implementation action evaluation team’s feedback influenced these changes. We consider these issues in the discussion that now follows.

### Conceptualizing Management Innovations Responding to an Emergency

Most IS studies have examined a tested change, planned and implemented over longer period of 1 year or more. Our study was of the fast implementation of an untested EMS, with many untested changes in the service units. Initially we used the Stages of Implementation Completion (SIC) framework to plan data gathering because it gave a way to document and represent each stage of the implementation of the EMS, and we also thought that we could use it to evaluate how well each stage was followed (Chamberlain et al., 2011).

However, as the pandemic continued longer than we expected, and the EMS was continually adapted, de-implemented and re-implemented in a modified form, we found the SIC framework to be more suited to the documentation of an unchanging innovation implementation. This conclusion was further supported by data in early 2021 about how the modified EMG and EMS implemented the vaccination program within SLSO. We found the SIC framework of limited use for both documenting and assisting the implementation of vaccinations, where it was necessary to manage a continually changing demand for and supply of vaccinations in different populations and communities. Instead, we found more useful for documenting adaptations to be the specifications in the framework initially proposed by Stirman, et al., (2013), and then further developed in the FRAME model (Wiltsey Stirman, et al., 2019) and applied empirically.

One IS study providing a framework closest to conceptualizing the data that showed the evolving and emergent characteristic of the EMS is the “dynamic sustainability framework” (DFS). This helps to consider the “fit between interventions, practice settings, and the broader ecological system over time” but also the dynamic nature of the changes (Chambers et al., 2013). However, applying this framework in practice proved difficult because we could not find a way to document, and to present clearly, the different changes at different times that were indicated in the DSF, even if we divided the documentation into periods relating to phases of the outbreak and context changes. Instead, we made more use of the above FRAME model. Also, the findings show data indicating the EMG trying different changes to their procedures and group methods as they “felt their way” toward implementing the EMS in the best way suited to the situation. Interviewees did not describe this iterative adaption as applying plan-do-study-act (PDSA) improvement cycles but as “like using PDSA.” Thus, we also found concepts from quality improvement useful to conceptualize the approach to implementing the EMS, and to consider how the implementation action evaluation team provided the EMG with data about the outbreak (Chambers, 2020; Langley et al., 2009).

### Implementing Innovations with No- or Uncertain- Evidence

A common question in implementation, and one frequently asked in discussions of responses to COVID-19 is, how certain or strong does the evidence of the effectiveness of an innovation need to be before using implementation methods to establish it in practice? Many management and organization innovation have not been rigorously tested in controlled trials and a number have not been tested at all (Kovner & D'Aunno, 2017; Walshe & Rundall, 2001). Some have been implemented elsewhere and there may be some reported experiential evidence from these implementations. In our study, neither the EMS nor many of the service delivery unit innovations had been tested in Stockholm.

In our project, we used the concept of “proportionate evidence” useful to conceptualize the findings about how the EMG made decisions about changes to the EMS and how they navigated the lack of or uncertain evidence about changes that they would direct SDU managers to implement. This concept is that the strength of evidence of effectiveness of an innovation required before implementation should be proportionate to the potential for harm, the likelihood of benefit, and the ease and cost of implementation (Øvretveit, 2014). For example, the innovation change of asking the receiver of an order placed over the telephone to read back the order: implementing this innovation change has a low potential for harm, a high likelihood of benefit, and the ease and cost of implementation is low. The management innovation of moving staff from one service with a low workload to a short-staffed service has a low potential for harm if they are trained, a likelihood of benefit, and the ease and cost of implementation is proportionate to the harm-to-benefit potential (Øvretveit, 2014).

If this proportionality principle is met, a second approach is to gather evidence of effectiveness of an innovation while implementing it. This can be carried out using established IS methods (Curran et al., 2012) or using an implementation action evaluation approach as described in this article.

### Limitations of an Implementation Action Evaluation Approach

Our collaborative and rapid response implementation study was possible because of a long partnership of mutual benefit and trust between the university and the P&CHc organization, and a team that could be quickly assembled composed of members from both organizations. The research team was unusual in combining researchers employed by the health system, with knowledge about and easy access to data bases and informants. The organization and management researchers were employed by a local medical university, and included some with joint appointments. This made it possible within two weeks to form a team, get ethics approval and start the weekly surveys using the internal secure staff information system. Partnership approaches are increasingly used in implementation science and proved essential for this study to achieve the dual objectives of rapid practical assistance and scientific contribution (Chambers & Azrin, 2013; Øvretveit et al., 2014, Øvretveit, 2002).

The collaborative implementation action evaluation approach, however, limits the generalization of the findings. The investigators regularly reported findings to the management team to enable them to improve the EMS, as well as to enable specific changes, such as distribution of personal protective equipment. The EMS innovation thus included researcher feedback about implementation progress. Others elsewhere may not have an investigation resource like this to enable iterative improvements, although many are moving toward a learning health system approach (Olsen et al., 2007). One approach is to make more use of theory and logic models to show the local configuration of elements resulting in outcomes (Damschroder, 2020). Another, perhaps imperfect, resolution is an assessment by independent investigators of how much this feedback changed the implementation and the innovation, for example by interviewing managers and implementers to find out their assessments (Waterman et al., 2001).

As a manager and an applied researcher, the authors propose the following would make IS studies, especially of scale up programs, more useful to others in other contexts and increase “external validity”: better descriptions of both the innovation (planned, and as operated in practice) and of the implementation methods (e.g., using the standard descriptions in the above-noted studies by, Powell et al., 2015; Michie et al., 2015; Stirman et al., 2013; Wiltsey Stirman et al., 2019). Also, comparable descriptions of the elements of context (e.g., using CFIR, 2021; Damschroder et al., 2009), and description of the people taking up the new better way of working and the population they are serving. Many of these points are covered in different guidelines for reporting research (Brownson et al., 2012). In addition, researchers could describe key principles that capture the most important aspect of the innovation and the implementation. They could provide guidance for interpreting principles locally and the data to collect to assess local effectiveness and safety, as well as or possible unintended negative consequences that need monitoring.

### Discussion Summary

This article joins with others in showing what implementation science and research can contribute to management and organization knowledge and practice in the future. Means et al. (2020), proposes that implementation science could enable more effective uptake of evidence-based innovations such as social distancing, hand washing, and PPE. Chambers (2020) also suggests using IS concepts to study de-implementation during and after the pandemic, as well as the effect of context, and draws attention to the importance of different levels aligning their communications and responses to emergencies. Some of the limitations described are, in part, because few studies have applied IS to management innovations, apart from studies in the field of quality and safety improvement (Morrow et al., 2012; Øvretveit, 2020b; Rubenstein et al., 2014).

## Conclusion

The team undertaking the study described here found some limitations to IS for providing practical rapid local evidence to managers in a rapidly changing situation to guide a primary and community healthcare organization to respond to a novel infectious disease. These included how to report the dynamic and fast evolution of an emergency management system, as well as generalizing the findings to be of use to others whose context may be different but critical to the implementation and operation of such an emergency system.

Our research did, however, find many concepts and methods developed by IS to be useful, and these could contribute to future emergency management research and to wider practice and research into implementation and health management and organization. Implementation research has found factors which help and hinder establishing new more effective practices and organization models, and these frameworks were useful to structure data collection in the study described. IS designs and methods helped us to focus data collection on the intermediate outcomes possibly related to the implementation of the EMS and on which elements of a changing context might influence the innovation and its implementation. Given the limitations of a time-series, single-case implementation action evaluation with no controls, we could only note the changes in intermediate outcomes that were reported by some managers interviewed and in survey responses to be due to the implementation of the EMS.

The pandemic showed that effective, and evidence-based implementation strategies are important for saving lives, reducing suffering and avoiding waste: implementation of vaccination is one example. There will be more emergencies in the future and some will be extended and evolving in unpredictable ways. We will need to use what we have, and to develop the potential for IS to contribute to fast implementation and testing of management innovations for emergencies and to developing this new body of knowledge and rapid impact research practice.

## Data Availability

Data from surveys, interviews and documents referred to in the article are available from the corresponding author, if time is allowed to remove information potentially identifying individuals and the purpose of the request is to verify the data reported.
